# Synthesis, crystal structure and Hirshfeld surface of bis­(2-amino­pyridinium) hexa­chlorido­stannate(IV)

**DOI:** 10.1107/S205698902000941X

**Published:** 2020-07-17

**Authors:** Rochdi Ghallab, Mehdi Boutebdja, George Dénès, Hocine Merazig

**Affiliations:** a Environmental Molecular, and Structural Chemistry Research Unit, University of, Constantine-1, 25000, Constantine, Algeria; b Laboratory of Solid State Chemistry and Mossbauer Spectroscopy, Departement of, Chemistry and Biochemistry, Concordia University, 7141 Sherbrooke St. West, Montreal, H4B 1R6, QC, Canada

**Keywords:** Zero-dimensional hybrid perovskite, amino­pyridine, Hirshfeld surface, Raman spectroscopy, crystal structure

## Abstract

In the title mol­ecular salt, (C_5_H_7_N_2_)_2_[SnCl_6_], the cation is protonated at the pyridine N atom and the complete dianion is generated by a crystallographic centre of symmetry. In the crystal, N—H⋯Cl hydrogen bonds link the components into a three-dimensional network built up from the stacking of alternate cationic and anionic layers. The nature of the inter­molecular inter­actions has been analysed in terms of the Hirshfeld surfaces of the cations and the anions. The thermal behaviour and the Raman spectrum of the title compound are reported.

## Abstract 2

The packing of the title mol­ecular salt, in which the tin atom lies on a crystallographic inversion centre, is dominated by N—H⋯Cl hydrogen bonds.

## Chemical context   

So-called ‘zero-dimensional’ hybrid perovskites are characterized by a structure formed by isolated inorganic octa­hedra (or bi­octa­hedra) and an organic cation (Cheng & Lin, 2010 ▸). They are easy to prepare through simple techniques (Mitzi, 2004 ▸) and they combine the properties of the various organic and inorganic compounds, *i.e*. the flexibility of the organic part, and the thermal stability and the rigidity of the inorganic part, in a single material, by cooperative effects, to obtain properties that are more than just the sum of the initial properties: an organic/inorganic ‘synergy’ is created. For example, in these hybrid materials, the organic part can have non-linear optical properties (Bi *et al.*, 2008 ▸). Most of the physical properties come from the inorganic part, such as the electronic transport properties, the optical photoluminescence properties (Yangui *et al.*, 2019 ▸), or even magnetic properties (Manser *et al.*, 2016 ▸). As part of our studies in this area, we now describe the synthesis and structure of the title mol­ecular salt, (I).
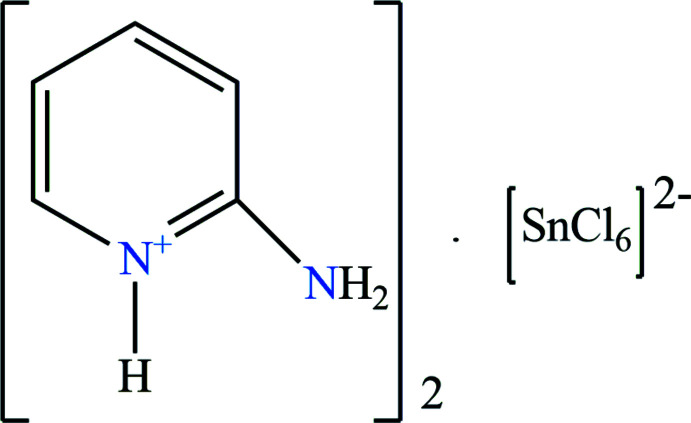


## Structural commentary   

**Figure 1:**
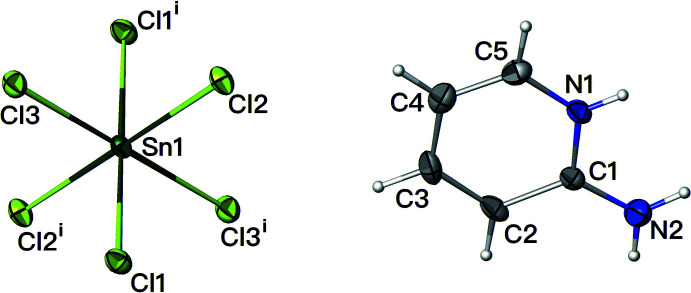
The mol­ecular structure of the title compound with displacement ellipsoids drawn at the 30% probability level [Symmetry code: (i) −*x*, −*y*, −*z*].

Compound (I) with formula (C_5_H_7_N_2_)^+^_2_·[SnCl_6_]^2−^, crystallizes in the triclinic space group *P* 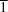 (Fig. 1 ▸).

In the synthesis, the oxidation number of tin changes from +2 to +4 such that the resultant tin(IV) atom is hexa­coordinated by chlorine atoms, generating a weakly distorted octa­hedron in which the metal ion lies on a crystallographic inversion centre: the length of the Sn—Cl bonds varies from 2.4216 (4) to 2.4474 (5) Å. As for the Cl—Sn—Cl angles, the discrepancy of about ± 1° [89.109 (18)–90.805 (16)°] compared to the 90° value angle of a regular octa­hedron shows that the angular distortion is very small. These values are comparable to those of the same anion associated with other types of cations (BelhajSalah *et al.*, 2018 ▸). The absence of larger distortions can probably be attributed to the fact that the hexa­chloro­stannate(IV) anions are free, *i.e*. none of the chloride ions are bridging although they do accept N—H⋯Cl hydrogen bonds from the organic cations, which ensures charge balance.

In the pyridinium ring of the cation, the C—C bond lengths vary from 1.328 (3) to 1.405 (3) Å and the C—N bond lengths are 1.341 (3) Å and 1.344 (2) Å. The values of the C—C—C angles in the pyridinium ring vary from 118.9 (2) to 120.9 (2)° whereas the C—N—C angle is 124.30 (18)°: the larger angle can be attributed to the protonation of the N atom. These values are comparable with those of the same cation associated with other types of anions (Rao *et al.*, 2011 ▸).

## Supra­molecular features   

**Figure 2:**
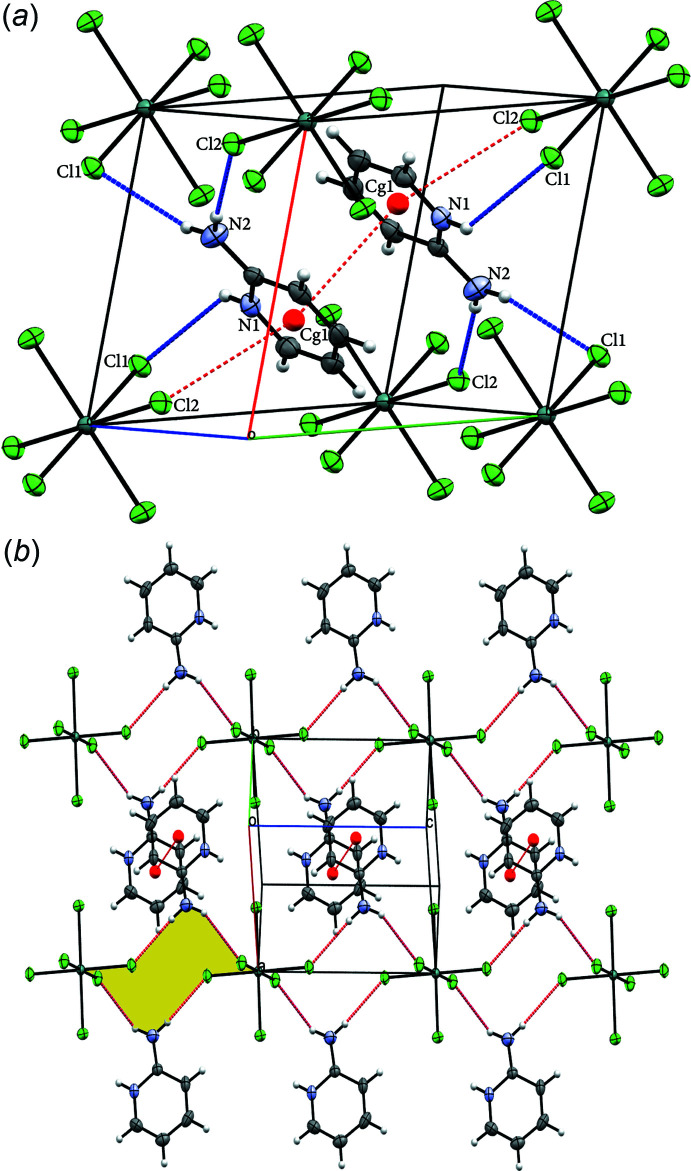
(*a*) Hydrogen bonds [symmetry codes: (i) *x*, *y* + 1, *z*; (ii) −*x* − 1, −*y* + 1, −*z*; (iii) −*x* − 1, −*y* + 1, −*z* + 1] (blue dashed lines), π-stacking inter­actions (symmetry operation: −1 − *x*, 1 − *y*, 1 − *z*) and Y—*X*⋯*Cg* (symmetry operation: *x*, −1 + *y*, *z*) (red dashed lines) in the unit cell of compound (I). (*b*) A view of the ring motifs along the *c* axis with the strongest hydrogen bonds and π-stacking inter­actions indicated by red dotted lines.

The special position of tin(IV) in the crystal of (I) gives rise to an alternation of cationic and anionic layers lying parallel to the (001) plane (Fig. 2 ▸ *a*). The inter­molecular inter­actions in (I) were analysed using *PLATON* (Spek, 2020 ▸), which shows that the structural cohesion in the crystalline structure of the compound (I) is ensured by N—H⋯Cl hydrogen bonds: Fig. 2 ▸ *a*. The distances and the angles describing these inter­actions are presented in Table 1 ▸.

The combination of N2—H2*A*⋯Cl1 and N2—H2*B*⋯Cl2 hydrogen bonds generates a chain of rings propagating along the [001] direction with a graph-set pattern of 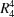
(12) (Etter *et al.*, 1990 ▸), Fig. 2 ▸(*b*). The cohesion between chains is ensured by π-stacking inter­actions between centrosymmetrically related aromatic rings of the cations: *Cg*1⋯*Cg*1 = 3.552 (13) Å; inter-planar angle α = 0.0 (11)°; slippage = 1.246 Å. We also note the presence of a *Y*—*X*⋯*Cg*1 type inter­action between Sn1—Cl2 and *Cg*1 at an *X*⋯*Cg* distance of 3.6581 (11) Å [Fig. 2 ▸(*a*)].

## Hirshfeld surface analysis:   

To further characterize the inter­molecular inter­actions in (I), the Hirshfeld surface method was used (Spackman & Jayatilaka, 2009 ▸). In addition, its two-dimensional fingerprints (Spackman & McKinnon, 2002 ▸) were calculated using the program *Crystal Explorer 17* (Turner *et al.*, 2017 ▸). The *d*_norm_ representation mode was used in which red spots identify close contacts; in the white areas, the distance separating the neighboring atoms approaches the sum of the van der Waals radii of the concerned atoms whereas blue areas illustrate areas where neighbouring atoms are too far apart to inter­act significantly with each other. The presence of the adjacent red and blue triangles, obtained by using the shape index as a representation mode, demonstrates the presence of π–π and *Y*—*X*⋯π type inter­actions.

**Figure 3:**
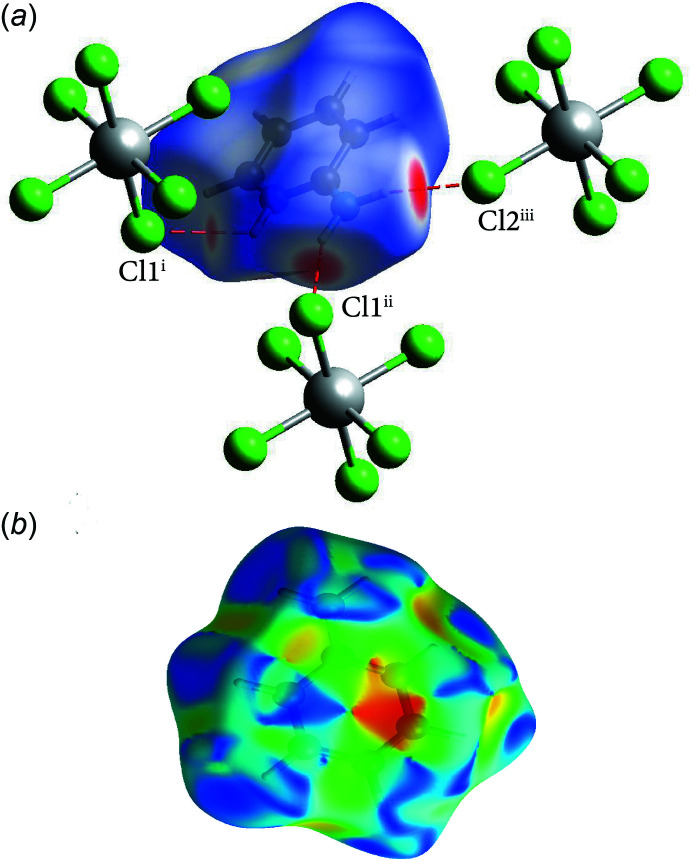
(*a*) A view of the Hirshfeld surface mapped over *d*_norm_ for compound (I) in the range −0.3387 to +1.0913 arbitrary units, highlighting the N—H⋯Cl inter­actions and (*b*) the Hirshfeld surface of the cation mapped over shape-index in the range −1.00 to +1.00 arbitrary units.

**Figure 4:**
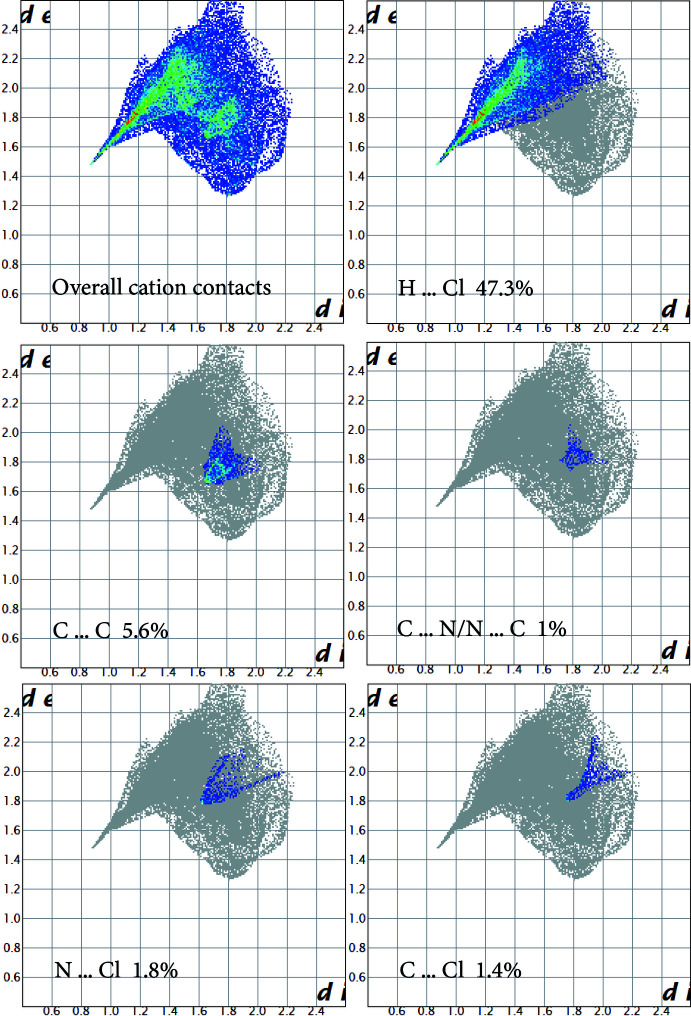
Two-dimensional fingerprint plots for the cation of the title compound, and delineated into the principal contributions of H⋯Cl, C⋯C, C⋯N/N⋯C, N⋯Cl and C⋯Cl contacts. Other significant contacts are H⋯H (21.1%), H⋯C/C⋯H (15.6%) and H⋯N/N⋯H (6.2%).

**Figure 5:**
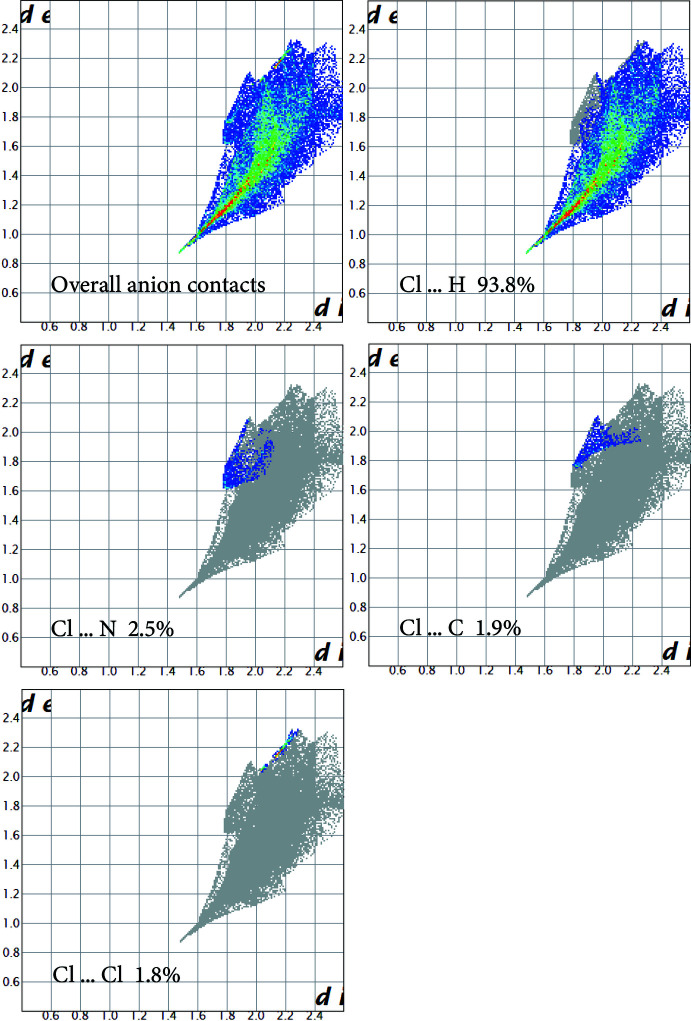
Two-dimensional fingerprint plots for the anion of the title compound, and those delineated into the principal contributions of Cl⋯H, Cl⋯C, Cl⋯N and Cl⋯Cl contacts.

The Hirshfeld surface [Fig. 3 ▸(*a*)] shows red spots corresponding to H⋯Cl/Cl⋯H close contacts, which are due to the N—H⋯Cl hydrogen bonds. The presence of the adjacent red and blue triangles in Fig. 3 ▸(*b*) demonstrates the presence of the *Cg*1⋯*Cg*1 and Sn—Cl2⋯*Cg*1 inter­actions. The contribution of different kinds of inter­atomic contacts to the Hirshfeld surfaces of the individual cations and anions is shown in the fingerprint plots in Fig. 4 ▸ and Fig. 5 ▸, respectively. These inter­actions are ensured by 47.3% of hydrogen bonds (H⋯Cl), 3.2% of *Y*—*X*⋯ type (N⋯Cl and C⋯Cl), 6.6% of π–π stacking type (C⋯C and C⋯N/N⋯C), 15.6% of C—H⋯π type (C⋯H/H⋯C), 6.2% of N—H⋯π type (N⋯H/H⋯N) and 21.1% of H⋯H van der Waals inter­actions. The two-dimensional fingerprint analysis for the anionic moieties reveals that hydrogen bonds (Cl⋯H) represent 93.8%, *Y*—*X*⋯π type inter­actions represent 4.4% (Cl⋯N and Cl⋯C) and van der Waals inter­actions of the Cl⋯Cl type represent 1.8% of the surface contacts.

## Database survey   

A search of the Cambridge Structural Database (CSD Version 5.41; Groom *et al.*, 2016 ▸) for structures similar to (I) gave several compounds such as 2-amino­pyridinium hexa­chloro­bis­muth(III) (Rao *et al.*, 2011 ▸), 2-amino­pyridinium hexa­chloro­indium(III) (Jin *et al.*, 2011 ▸), 4-amino­pyridinium hexa­chloro­anti­monate(V) (Kulicka *et al.*, 2006 ▸) and 4-amino­pyridinium hexa­chloro­stannate(IV) (Rademeyer *et al.*, 2007 ▸) among others, but the last of these (refcode RIGDER) is of particular inter­est: RIGDER and (I) both crystallize in space group *P* 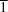 where the [SnCl_6_]^2−^ anions are associated with special positions and an organic–inorganic layered structure lying parallel to the (001) plane results.

Crystalline cohesion in RIGDER and (I) is ensured by dipole–dipole inter­actions and hydrogen bonds of the N—H⋯Cl type with a slight difference in the donor–acceptor angles and distances of the two compounds. The different arrangement of the nitro­gen atoms in the cation in RIGDER leads to much weaker π–π stacking compared to (I): the centroid separations are 4.24 (1) and 3.552 (13) Å, respectively. We also notice a slight difference between the two compounds in the inter­action percentages calculated by the Hirshfeld surface analysis (see Table S1 in the supporting information).

## Thermal analysis   

In order to investigate the thermal stability of (I), thermogravimetric analysis (DTA/TGA) was performed under an N_2_ atmosphere at a heating rate of 10°C min^−1^ in the temperature range from 25 to 500°C. The thermogram of (I) (see Fig. S2 in the supporting information) shows that the compound loses 64.4% of its mass in the temperature range of 270–304°C. The mass loss can be attributed to the degradation of the organic entity and two chlorine atoms (Janiak & Blazejowski, 1990 ▸) to leave a reside of SnCl_4_.

## Synthesis and crystallization   

Tin(II) chloride dihydrate (2.25 mmol) was mixed with 2-amino­pyridine (0.94 mmol) and a few drops of hydro­chloric acid in an aliquot of distilled water in 1:1 molar ratio was added. After stirring, the mixture was poured into a vial (biotage microwave vial 2–5 ml) that was put in an oven for three days at 393 K. Upon cooling, prism-shaped crystals of (I) were obtained and separated using an optical microscope. ^1^H NMR (δ ppm), 400 MHz, CDCl_3_): 8.16 (*br s*, 2H, NH_2_), 7.95–7.89 (*m*, 2H CH Py), 7.03 (*d*, *J*_HH_ = 8.9 Hz,1H CH Py), 6.84 (*t*, *J*_HH_ = 6.6 Hz, 1H CH Py). ^13^C NMR (δ ppm), 125 MHz, CDCl_3_): 154.6 (quat C Py), 144.4 (CH Py), 136.1 (CH Py), 113.8 (CH Py), 112.5 (CH Py).

**Figure 6:**
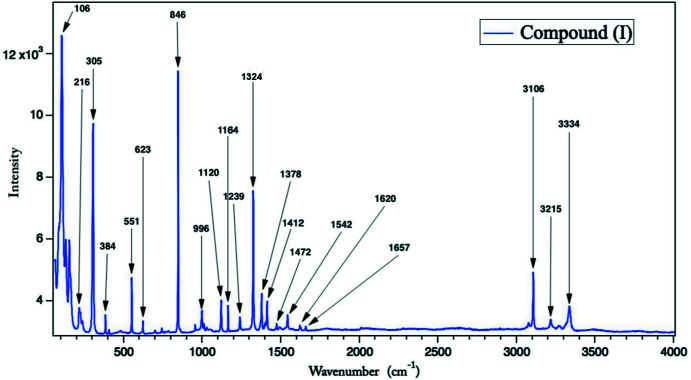
Raman spectrum of compound (I).

The raman spectrum for (I) (Fig. 6 ▸) was recorded in the frequency range 4000–60 cm^−1^. The Py, ν, δ, γ and τ are: pyridine ring, stretching, in-plane bending, out-of-plane bending and torsion, respectively. RS (cm^−1^): 3334 ν(N—H), 3215 ν (N—H), 3106 ν(C—H), 1657 ν(py)+δ(N—H)+δ(NH_2_), 1620 ν(py), 1542 ν(py), 1472 ν(py)+δ(C—H), 1412 ν(py)+δ(C—H), 1378 ν(py)+δ(C—H), 1324 ν(py)+δ(C—H), 1239 ν(py)+δ(C—H), 1164 δ(py)+δ(C—H), 1120 δ(py)+δ(C—H), 996 δ(py), 846 Pyridine ring breathing+γ(C—H), 623 γ(py), 551 γ(py), 406 γ(py), 384 γ(py), 305 ν_1_ (Sn—Cl), 216 ν_2_ (Sn—Cl), 106 τ(py)+ ν(N—H⋯Cl) (Shaw *et al.*, 1988 ▸; Ureña *et al.*, 2003 ▸; Cook, 1961 ▸).

## Refinement   

Crystal data, data collection and structure refinement details are summarized in Table 2 ▸. The C-bound H atoms and the anine H atom were placed geometrically and refined as riding atoms [C—H = 0.93 Å and *U*_iso_(H) = 1.2*U*_eq_(C)]; the pyridine N—H atom was located in a difference map and its position was freely refined.

## Supplementary Material

Crystal structure: contains datablock(s) I. DOI: 10.1107/S205698902000941X/hb7922sup1.cif

Structure factors: contains datablock(s) I. DOI: 10.1107/S205698902000941X/hb7922Isup2.hkl

Supporting information file. DOI: 10.1107/S205698902000941X/hb7922sup3.jpg

Supporting information file. DOI: 10.1107/S205698902000941X/hb7922sup4.docx

CCDC reference: 1904730

Additional supporting information:  crystallographic information; 3D view; checkCIF report

## Figures and Tables

**Table 1:** Hydrogen-bond geometry (Å, °)

*D*—H⋯*A*	*D*—H	H⋯*A*	*D*⋯*A*	*D*—H⋯*A*
N1—H1⋯Cl1^i^	0.77 (2)	2.79 (3)	3.3155 (18)	128 (3)
N2—H2*A*⋯Cl1^ii^	0.86	2.62	3.406 (2)	152
N2—H2*B*⋯Cl2^iii^	0.86	2.52	3.358 (2)	166

Symmetry codes: (i) 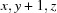; (ii) 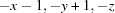; (iii) 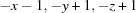.

**Table 2:** Experimental details

Crystal data	
Chemical formula	(C_5_H_7_N_2_)_2_[SnCl_6_]
*M*_r_	521.64
Crystal system, space group	Triclinic, *P* 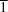
Temperature (K)	298
*a*, *b*, *c* (Å)	7.4537 (1), 8.0674 (1), 8.1025 (1)
α, β, γ (°)	83.791 (1), 82.591 (1), 71.407 (1)
*V* (Å^3^)	456.77 (1)
*Z*	1
Radiation type	Mo *K*α
μ (mm^−1^)	2.27
Crystal size (mm)	0.08 × 0.08 × 0.07

Data collection	
Diffractometer	Bruker SMART APEXII area detector
Absorption correction	Multi-scan (*SADABS*; Bruker, 2016 ▸)
*T*_min_, *T*_max_	0.834, 0.853
No. of measured, independent and observed [*I* > 2σ(*I*)] reflections	10455, 2011, 1910
*R*_int_	0.021
(sin θ/λ)_max_ (Å^−1^)	0.641

Refinement	
*R*[*F* ^2^ > 2σ(*F* ^2^)], *wR*(*F* ^2^), *S*	0.017, 0.037, 1.06
No. of reflections	2011
No. of parameters	101
No. of restraints	36
H-atom treatment	H atoms treated by a mixture of independent and constrained refinement
Δρ_max_, Δρ_min_ (e Å^−3^)	0.56, −0.34

Computer programs: *APEX2* and *SAINT* (Bruker, 2016 ▸), *SIR2004* (Burla *et al.*, 2007 ▸), *SHELXL* (Sheldrick, 2015 ▸) and *OLEX2* (Dolomanov *et al.*, 2009 ▸).

## supplementary crystallographic information

### Crystal data

**Table:**

2C_5_H_7_N_2_^+^·Cl_6_Sn^2^^−^	*Z* = 1
*M**_r_* = 521.64	*F*(000) = 254
Triclinic, *P*1	*D*_x_ = 1.896 Mg m^−^^3^
*a* = 7.4537 (1) Å	Mo *K*α radiation, λ = 0.71073 Å
*b* = 8.0674 (1) Å	Cell parameters from 1910 reflections
*c* = 8.1025 (1) Å	θ = 3.6–27.1°
α = 83.791 (1)°	µ = 2.27 mm^−^^1^
β = 82.591 (1)°	*T* = 298 K
γ = 71.407 (1)°	Prism, colourless
*V* = 456.77 (1) Å^3^	0.08 × 0.08 × 0.07 mm

### Data collection

**Table:**

Bruker SMART APEXII area detector diffractometer	2011 independent reflections
Radiation source: microfocus sealed X-ray tube, Incoatec Iµs	1910 reflections with *I* > 2σ(*I*)
Mirror optics monochromator	*R*_int_ = 0.021
Detector resolution: 7.9 pixels mm^-1^	θ_max_ = 27.1°, θ_min_ = 3.6°
ω and φ scans	*h* = −9→9
Absorption correction: multi-scan (SADABS; Bruker, 2016)	*k* = −10→10
*T*_min_ = 0.834, *T*_max_ = 0.853	*l* = −10→10
10455 measured reflections	

### Refinement

**Table:**

Refinement on *F*^2^	Primary atom site location: structure-invariant direct methods
Least-squares matrix: full	Hydrogen site location: mixed
*R*[*F*^2^ > 2σ(*F*^2^)] = 0.017	H atoms treated by a mixture of independent and constrained refinement
*wR*(*F*^2^) = 0.037	*w* = 1/[σ^2^(*F*_o_^2^) + (0.0121*P*)^2^ + 0.2078*P*] where *P* = (*F*_o_^2^ + 2*F*_c_^2^)/3
*S* = 1.06	(Δ/σ)_max_ < 0.001
2011 reflections	Δρ_max_ = 0.56 e Å^−^^3^
101 parameters	Δρ_min_ = −0.33 e Å^−^^3^
36 restraints	

### Special details

**Table:**

Geometry. All esds (except the esd in the dihedral angle between two l.s. planes) are estimated using the full covariance matrix. The cell esds are taken into account individually in the estimation of esds in distances, angles and torsion angles; correlations between esds in cell parameters are only used when they are defined by crystal symmetry. An approximate (isotropic) treatment of cell esds is used for estimating esds involving l.s. planes.

### Fractional atomic coordinates and isotropic or equivalent isotropic displacement parameters (Å^2^)

**Table:**

	*x*	*y*	*z*	*U*_iso_*/*U*_eq_	
Sn1	0.000000	0.000000	0.000000	0.03137 (6)	
Cl3	−0.29282 (7)	0.24458 (7)	−0.00146 (6)	0.04838 (12)	
Cl1	−0.17021 (7)	−0.18776 (7)	−0.08097 (6)	0.04442 (11)	
Cl2	−0.07788 (7)	−0.07356 (7)	0.29243 (5)	0.04473 (12)	
C1	−0.4688 (3)	0.7533 (2)	0.4088 (2)	0.0364 (4)	
C2	−0.4174 (3)	0.6744 (3)	0.5662 (2)	0.0455 (5)	
H2	−0.481358	0.726282	0.663052	0.055*	
C3	−0.2718 (3)	0.5200 (3)	0.5747 (3)	0.0538 (6)	
H3	−0.237750	0.465902	0.678377	0.065*	
C4	−0.1736 (3)	0.4425 (3)	0.4298 (3)	0.0540 (5)	
H4	−0.075494	0.337051	0.436149	0.065*	
C5	−0.2228 (3)	0.5220 (3)	0.2827 (3)	0.0498 (5)	
H5	−0.157176	0.473379	0.184945	0.060*	
N1	−0.3668 (3)	0.6723 (2)	0.2748 (2)	0.0414 (4)	
N2	−0.6083 (3)	0.9016 (2)	0.3872 (3)	0.0568 (5)	
H2A	−0.633148	0.945458	0.288064	0.068*	
H2B	−0.674139	0.954202	0.472330	0.068*	
H1	−0.397 (4)	0.716 (3)	0.189 (3)	0.061 (8)*	

### Atomic displacement parameters (Å^2^)

**Table:**

	*U*^11^	*U*^22^	*U*^33^	*U*^12^	*U*^13^	*U*^23^
Sn1	0.02900 (9)	0.04010 (10)	0.02432 (9)	−0.01029 (7)	−0.00234 (6)	−0.00090 (6)
Cl3	0.0391 (2)	0.0533 (3)	0.0432 (3)	0.0020 (2)	−0.0084 (2)	−0.0073 (2)
Cl1	0.0495 (3)	0.0531 (3)	0.0382 (2)	−0.0260 (2)	−0.0058 (2)	−0.0026 (2)
Cl2	0.0467 (3)	0.0600 (3)	0.0261 (2)	−0.0177 (2)	0.00014 (18)	0.00261 (19)
C1	0.0335 (9)	0.0424 (10)	0.0374 (9)	−0.0171 (8)	−0.0039 (7)	−0.0036 (8)
C2	0.0521 (12)	0.0664 (13)	0.0283 (9)	−0.0338 (11)	−0.0015 (8)	−0.0027 (8)
C3	0.0577 (13)	0.0643 (14)	0.0501 (12)	−0.0345 (12)	−0.0251 (10)	0.0215 (10)
C4	0.0444 (12)	0.0477 (12)	0.0717 (15)	−0.0166 (10)	−0.0112 (10)	0.0014 (10)
C5	0.0458 (11)	0.0468 (12)	0.0570 (13)	−0.0158 (10)	0.0039 (10)	−0.0114 (10)
N1	0.0500 (10)	0.0482 (10)	0.0288 (8)	−0.0199 (8)	−0.0027 (7)	−0.0015 (7)
N2	0.0529 (11)	0.0540 (11)	0.0573 (11)	−0.0035 (9)	−0.0117 (9)	−0.0105 (9)

### Geometric parameters (Å, º)

**Table:**

Sn1—Cl3^i^	2.4315 (5)	C2—C3	1.369 (3)
Sn1—Cl3	2.4315 (5)	C3—H3	0.9300
Sn1—Cl1	2.4474 (4)	C3—C4	1.397 (3)
Sn1—Cl1^i^	2.4474 (4)	C4—H4	0.9300
Sn1—Cl2^i^	2.4216 (4)	C4—C5	1.328 (3)
Sn1—Cl2	2.4216 (4)	C5—H5	0.9300
C1—C2	1.405 (3)	C5—N1	1.341 (3)
C1—N1	1.344 (2)	N1—H1	0.77 (2)
C1—N2	1.324 (3)	N2—H2A	0.8600
C2—H2	0.9300	N2—H2B	0.8600
			
Cl3^i^—Sn1—Cl3	180.0	C1—C2—H2	120.6
Cl3^i^—Sn1—Cl1	90.889 (18)	C3—C2—C1	118.89 (19)
Cl3—Sn1—Cl1^i^	90.891 (18)	C3—C2—H2	120.6
Cl3—Sn1—Cl1	89.109 (18)	C2—C3—H3	119.6
Cl3^i^—Sn1—Cl1^i^	89.111 (18)	C2—C3—C4	120.85 (19)
Cl1^i^—Sn1—Cl1	180.0	C4—C3—H3	119.6
Cl2—Sn1—Cl3^i^	89.575 (17)	C3—C4—H4	120.6
Cl2^i^—Sn1—Cl3^i^	90.425 (17)	C5—C4—C3	118.9 (2)
Cl2^i^—Sn1—Cl3	89.575 (17)	C5—C4—H4	120.6
Cl2—Sn1—Cl3	90.425 (17)	C4—C5—H5	119.9
Cl2—Sn1—Cl1	90.805 (16)	C4—C5—N1	120.1 (2)
Cl2^i^—Sn1—Cl1^i^	90.804 (16)	N1—C5—H5	119.9
Cl2^i^—Sn1—Cl1	89.196 (16)	C1—N1—H1	116.4 (19)
Cl2—Sn1—Cl1^i^	89.195 (16)	C5—N1—C1	124.30 (18)
Cl2^i^—Sn1—Cl2	180.0	C5—N1—H1	119.3 (19)
N1—C1—C2	116.94 (18)	C1—N2—H2A	120.0
N2—C1—C2	123.55 (18)	C1—N2—H2B	120.0
N2—C1—N1	119.50 (18)	H2A—N2—H2B	120.0

Symmetry code: (i) −*x*, −*y*, −*z*.

### Hydrogen-bond geometry (Å, º)

**Table:**

*D*—H···*A*	*D*—H	H···*A*	*D*···*A*	*D*—H···*A*
N1—H1···Cl1^ii^	0.77 (2)	2.79 (3)	3.3155 (18)	128 (3)
N2—H2*A*···Cl1^iii^	0.86	2.62	3.406 (2)	152
N2—H2*B*···Cl2^iv^	0.86	2.52	3.358 (2)	166

Symmetry codes: (ii) *x*, *y*+1, *z*; (iii) −*x*−1, −*y*+1, −*z*; (iv) −*x*−1, −*y*+1, −*z*+1.
