# Supplementary figures and images for: Synthesis, crystal structure and Hirshfeld surface of bis­(2-amino­pyridinium) hexa­chlorido­stannate(IV)
Source: Acta Crystallogr E Crystallogr Commun. 2020 Jul 17;76(Pt 8):1279–83. doi: 10.1107/S205698902000941X (PMC7405591; doi:10.1107/S205698902000941X)

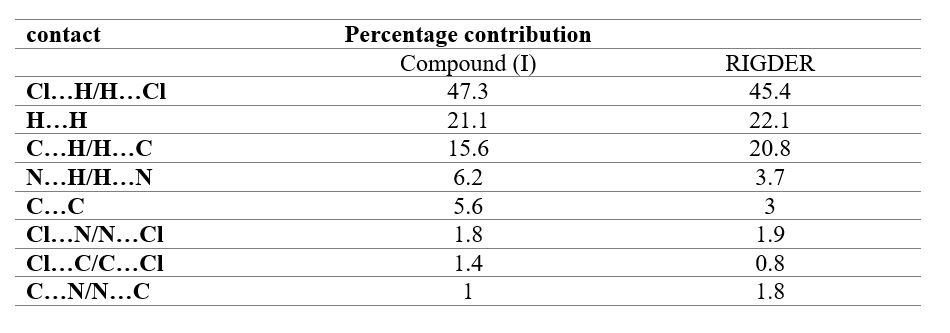

Supplement: Supplementary file 3 [file e-76-01279-sup3.jpg]

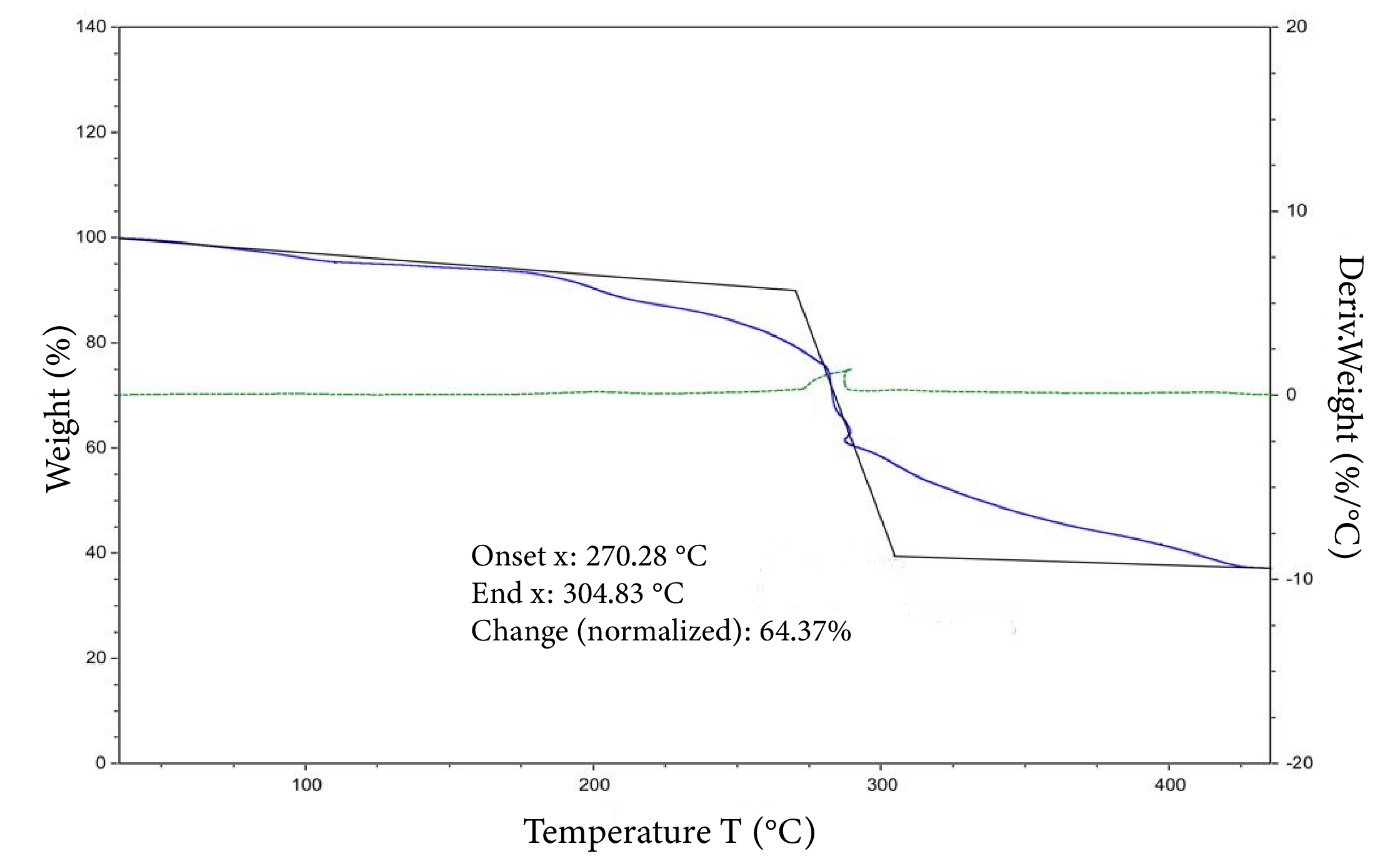

Supplement: Supplementary file 4 [file e-76-01279-sup4.docx]
